# Prevalence and co-prevalence of comorbidities among Chinese adult patients with type 2 diabetes mellitus: a cross-sectional, multicenter, retrospective, observational study based on 3B study database

**DOI:** 10.3389/fendo.2024.1362433

**Published:** 2024-06-11

**Authors:** Qiuhe Ji, Shangyu Chai, Ruya Zhang, Jihu Li, Yiman Zheng, Swapnil Rajpathak

**Affiliations:** ^1^ Department of Endocrinology and Metabolism, Xi’an International Medical Center Hospital, Shanxi, China; ^2^ Value & Implementation Global Medical & Scientific Affairs, Merck Sharp & Dohme (MSD) China, Shanghai, China; ^3^ Government Affairs & Market Access, Merck Sharp & Dohme (MSD) China, Shanghai, China; ^4^ Value & Implementation Outcomes Research, Merck Research Laboratories, Merck & Co., Inc., Rahway, NJ, United States

**Keywords:** type 2 diabetes, comorbidity, prevalence, co-prevalence, 3B study

## Abstract

**Purpose:**

This study aimed to investigate the prevalence and co-prevalence of comorbidities among Chinese individuals with type 2 diabetes (T2DM).

**Methods:**

Medical records were retrospectively retrieved from the 3B Study database, which provided a comprehensive assessment of comorbid conditions in Chinese adult outpatients with T2DM. Patient characteristics, laboratory measures, and comorbidities were summarized via descriptive analyses, overall and by subgroups of age (<65, 65–74, 75 years) and gender.

**Results:**

Among 25,454 eligible patients, 53% were female, and the median age was 63 years. The median time of diabetes duration was 6.18 years. A total of 20,309 (79.8%) patients had at least one comorbid condition alongside T2DM. The prevalence of patients with one, two, three, and four or more comorbid conditions was 28.0%, 24.6%, 15.6%, and 11.6%, respectively. Comorbidity burden increased with longer T2DM duration. Older age groups also exhibited higher comorbidity burden. Females with T2DM had a higher overall percentage of comorbidities compared to males (42.7% vs. 37.1%). The most common comorbid conditions in T2DM patients were hypertension (HTN) in 59.9%, overweight/obesity in 58.3%, hyperlipidemia in 42.0%, retinopathy in 16.5%, neuropathy in 15.2%, cardiovascular disease (CVD) in 14.9%, and renal disease in 14.4%. The highest co-prevalence was observed for overweight/obesity and HTN (37.6%), followed by HTN and hyperlipidemia (29.8%), overweight/obesity and hyperlipidemia (27.3%), HTN and CVD (12.6%), HTN and retinopathy (12.1%), and HTN and renal disease (11.3%).

**Conclusion:**

The majority of T2DM patients exhibit multiple comorbidities. Considering the presence of multimorbidity is crucial in clinical decision-making.

**Systematic review registration:**

https://clinicaltrials.gov/, identifier NCT01128205.

## Introduction

Diabetes, with over 90% of cases being type 2 diabetes (T2DM), stands as a significant global public health concern, exerting an escalating burden worldwide (1). The International Diabetes Federation reported a staggering 537 million affected adults globally in 2021, a number projected to ascend to 643 million by 2030 and a daunting 784 million by 2045 (2). China mirrors this upward trend, holding the highest number of diabetic patients globally. A recent prevalence of diabetes in China accounted for 10.6% to 12.8% of the total population, corresponding to approximately 141 million individuals in 2021 (2–4). Furthermore, with the incidence of DM continuing to escalate, this figure is expected to significantly increase to 174.4 million by 2045 (2–4). Moreover, alarming statistics revealed that 15.5% to 50.1% of Chinese adults are struggling with pre-diabetes (5, 6). This reality exerts immense pressure on healthcare practitioners in China, particularly those specializing in the care of T2DM patients.

T2DM patients frequently have complex comorbidities (7–10). A large-scale comprehensive study in the USA indicated that a staggering 97.5% of T2DM patients exhibited at least one comorbidity, with 88.5% dealing with two or more (8). In comparable western nations, 82% to 95.0% of T2DM patients grappled with multiple comorbid conditions (9, 10). Guidelines from the American Diabetes Association (ADA, 2023) and the Chinese Diabetes Society (CDS, 2020) stress personalized management considering comorbidities such as atherosclerotic cardiovascular disease (CVD), obesity, hypertension (HTN), hyperlipidemia and chronic kidney disease (CKD) (11, 12). An integrated and comprehensive evaluation of concurrent conditions enhances T2DM management, thereby elevating patients’ quality of life and reducing mortality risks (13, 14). However, current evidence remains inadequate in illuminating the prevalence and co-occurrence of comorbidities among Chinese T2DM patients. A limited-scale study conducted in Zhejiang province, China revealed that 93.7% of 4777 T2DM patients had comorbidities, averaging three conditions per individual (15). Still, the generalizability of this single-center study to the broader Chinese T2DM population is uncertain.

Hence, this cross-sectional study utilizes the existing 3B Study (National Assessment of Cardiovascular Risk Factors: Blood Glucose, Blood Pressure, and Blood Lipid in Chinese Patients with Type 2 Diabetes) database to elucidate the prevalence and co-occurrence of comorbidities, including HTN, hyperlipidemia, CVD, cerebrovascular disease (CBD), congestive heart failure (CHF), peripheral vascular diseases (PVD), renal disease, retinopathy, and neuropathy among Chinese T2DM patients (16). Age and sex-stratified analyses will also be conducted to provide further insights to the findings.

## Patients and methods

### Study design

This study was a cross-sectional, multicenter, retrospective, and observational investigation. The data for analysis were obtained from the 3B Study (Nationwide Assessment of Cardiovascular Risk Factors: Blood Glucose, Blood Pressure, and Blood Lipid in Chinese Patients with Type 2 Diabetes Mellitus) database, which comprised medical records of adult Chinese patients diagnosed with T2DM according to the World Health Organization criteria, as recommended by the Chinese Diabetes Society Guideline before 2020 (16–22). The 3B Study enrolled patients from cardiology, endocrinology, nephrology, and internal medicine clinics in a total of 104 hospitals, which were categorized into three tiers: community hospitals (Tier 1), secondary/city level hospitals (Tier 2), and teaching or comprehensive central hospitals (Tier 3). These hospitals were distributed across all major geographical regions in China, encompassing North China, South China, East China, West China, and Northeast China. This study was approved by the ethics committee of Peking University People’s Hospital and by ethics committees at other hospitals where an individual committee review was required (registered at clinicaltrials.gov, No. NCT01128205). All procedures performed in studies involving human participants were in accordance with the ethical standards of the institutional and/or national research committee and with the 1964 Helsinki Declaration and its later amendments or comparable ethical standards.

### Patients and data collection

Patients enrolled with a history of T2DM for at least 6 months and 18 years of age or older were included in this study. Patients with Type 1 DM in any of their previous records or pregnancy at enrollment were excluded.

Patients’ demographic features, residential location, education level, duration of T2DM, and laboratory measurements obtained within 30 days before or 7 days after screening were collected. Estimated GFR (eGFR) was calculated from the serum creatinine, age and sex. A smoker was defined as an individual who has smoked at least one cigarette per day for a minimum of one year, including both current and past smokers. A drinker was defined as someone who has consumed at least 50 grams of alcohol per day for a minimum of one year, including both current and past drinkers. Comorbidities either presented at the time of enrollment in the 3B study or recorded in their prior medical history (≥ 6 months) were collected. Among the comorbidities examined, HTN, hyperlipidemia, CVD, CBD, CHF, PVD, renal disease, overweight, obesity, retinopathy, and neuropathy were of interest. The researchers established definitions for each of these conditions based on clinical criteria. HTN was identified as either a present illness or a prior medical history of hypertension, with systolic blood pressure (SBP) ≥140 mmHg or diastolic blood pressure (DBP) ≥90 mmHg (23). Hyperlipidemia was defined by a low-density lipoprotein cholesterol (LDLc) ≥160 mg/dl using the most recent measure prior to the index date, or have a prior history of hyperlipidemia or use of medications for lowering cholesterol (24). Overweight and obesity were defined as BMI of 24–27.9 kg/m^2^ and ≥28.0 kg/m^2^ (25, 26). CVD included stable angina, unstable angina, myocardial infarction, percutaneous coronary intervention, or underwent coronary bypass. CBD included ischemic stroke, hemorrhagic stroke, or transient ischemic attack. PVD involved carotid artery diseases, intermittent claudication, diabetic foot and amputation. Renal disease included diabetic nephropathy and other chronic kidney diseases (CKD stage 3–5). Retinopathy included conditions such as glaucoma, cataracts, and blindness due to retinal damage. Neuropathy included nerve pain, and skin injuries.

### Statistical analysis

Patient characteristics, comorbidities, and laboratory measures were subjected to descriptive analyses to provide a comprehensive overview. Continuous variables were expressed as medians, including the interquartile range, while categorical variables were presented as the number and percentage of the total study population. Additionally, the percentages of patients with various combinations of pairs of comorbidities were calculated. The analyses were carried out for both the overall population and subgroups based on age (<65, 65–74, 75þ years) and gender. The two-sided 95% confidence interval (CI) was conducted by Wilson score method. Statistical analyses were performed using SAS version 9.2 or a higher version (SAS Institute, Cary, NC, USA).

## Results

### Characteristics of the study population

The baseline characteristics were displayed in **Table 1**. A total of 25,454 eligible patients were included in the study. The median age of the overall population was 63 years, with 53% of the patients being female. A significant majority (89.5%) of the patients lived in urban areas, while only 24.2% had received education beyond high school. The median duration of T2DM was 6.18 years, and 58% of the patients had been diagnosed with T2DM for over 5 years. Among the participants, 54.4% were under 65 years of age, 28.0% were aged between 65 and 74 years, and 17.4% were 75 years or older. The median age for males was 61 years, and for females, it was 64 years. Females tended to have a longer duration of T2DM compared to males, with median durations of 6.82 years and 5.81 years, respectively.

**Table 1:** Characteristics of the study population, overall, by age and gender.

	Overall		Age group						Gender			
			<65 years		65–74 years		≥ 75 years		Male		Female	
	N	Median (Q1-Q3) or %	N	Median (Q1-Q3) or %	N	Median (Q1-Q3) or %	N	Median (Q1-Q3) or %	N	Median (Q1-Q3) or %	N	Median (Q1-Q3) or %
**Patients enrolled**	25454		13855		7133		4421		11955		13499	
**Age (years)**	25409	63.0 (55.0–72.0)	13855	55.0 (49.0–60.0)	7133	70.0 (67.0–72.0)	4421	78.0 (76.0–81.0)	11932	61.0 (52.0–71.0)	13477	64.0 (56.0–72.0)
Gender												
Male	11955	47.0	7047	50.9	2982	41.8	1903	43.0	NA	NA	NA	NA
Female	13499	53.0	6808	49.1	4151	58.2	2518	57.0	NA	NA	NA	NA
Residence												
Urban	22776	89.5	11973	86.4	6565	92.0	4193	94.8	10885	91.0	11891	88.1
Rural	2678	10.5	1882	13.6	568	8.0	228	5.2	1070	9.0	1608	11.9
Education												
≤High school	19298	75.8	10019	72.3	5512	77.3	3729	84.3	7851	65.7	11447	84.8
>High school	6156	24.2	3836	27.7	1621	22.7	692	15.7	4104	34.3	2052	15.2
Duration of T2DM (years)												
Overall	25454	6.18 (2.67–11.31)	13855	4.98 (2.03–10.03)	7133	8.15 (3.59–13.42)	4421	9.48 (3.98–15.56)	11955	5.81 (2.43–10.93)	13499	6.82 (2.84–12.08)
<1	2294	9.0	1606	11.6	444	6.2	243	5.5	1223	10.2	1071	7.9
1–5	8397	33.0	5348	38.6	1965	27.5	1076	24.3	4121	34.5	4276	31.7
Laboratory Measure HbA1c (%)												
Overall	25411	7.10 (6.27–8.60)	13834	7.30 (6.30–8.90)	7121	7.00 (6.20–8.20)	4411	7.00 (6.20–8.19)	11933	7.20 (6.30–8.80)	13478	7.10 (6.20–8.40)
<7	11403	44.8	5805	41.9	3480	48.8	2090	47.3	5142	43.0	6261	46.4
7 to <8	5481	21.5	2807	20.3	1588	22.3	1080	24.4	2556	21.4	2925	21.7
8 to <9	3180	12.5	1790	12.9	864	12.1	522	11.8	1489	12.5	1691	12.5
≥9	5347	21.0	3432	24.8	1189	16.7	719	16.3	2746	23.0	2601	19.3
**SBP (mmHg)**	25453	130.00 (120.00–140.00)	13854	130.00 (120.00–140.00)	7133	130.00 (125.00–140.00)	4421	132.00 (125.00- 142.00)	11955	130.00 (120.00–140.00)	13498	130.00 (120.00–140.00)
**DBP (mmHg)**	25453	80.00 (70.00–82.00)	13854	80.00 (70.00–85.00)	7133	80.00 (70.00–80.00)	4421	78.00 (70.00- 80.00)	11955	80.00 (70.00–84.00)	13498	80.00 (70.00–80.00)
**Total cholesterol** **(mmol/L)**	25438	4.89 (4.15–5.67)	13845	4.96 (4.23–5.73)	7130	4.85 (4.10–5.65)	4418	4.70 (3.93–5.49)	11946	4.68 (3.97–5.43)	13492	5.08 (4.32–5.87)
**LDL-c (mmol/L)**	25399	2.76 (2.21–3.35)	13825	2.80 (2.24–3.41)	7118	2.74 (2.21–3.31)	4411	2.66 (2.11–3.25)	11925	2.68 (2.15–3.25)	13474	2.83 (2.28–3.45)
**HDL-c (mmol/L)**	25446	1.23 (1.02–1.50)	13850	1.22 (1.01–1.48)	7131	1.26 (1.04–1.51)	4420	1.24 (1.01–1.50)	11953	1.16 (0.96–1.40)	13493	1.30 (1.09–1.57)
**Triglycerides (mmol/L)**	25403	1.56 (1.10–2.27)	13818	1.63 (1.12–2.45)	7127	1.52 (1.10–2.15)	4413	1.42 (1.02–2.01)	11929	1.50 (1.04–2.25)	13474	1.60 (1.14–2.30)
eGFR												
Overall	22589	88.97 (67.02–113.03)	12265	99.41 (77.78–122.99)	6299	80.46(61.51–102.07)	4025	71.74 (53.96–92.99)	10752	92.50 (71.77–115.48)	11837	85.29 (62.81–110.29)
≥90	11025	43.3	7534	54.4	2384	33.4	1107	25.0	5716	47.8	5309	39.3
60 to <90	7429	29.2	3403	24.6	2462	34.5	1564	35.4	3483	29.1	3946	29.2
45 to <60	2279	9.0	696	5.0	835	11.7	748	16.9	812	6.8	1467	10.9
30 to <45	957	3.8	248	1.8	337	4.7	372	8.4	315	2.6	642	4.8
15 to <30	439	1.7	163	1.2	140	2.0	136	3.1	195	1.6	244	1.8
<15	460	1.8	221	1.6	141	2.0	98	2.2	231	1.9	229	1.7
BMI (kg/m^2^)												
<24	10617	41.7	5386	38.9	3155	44.2	2051	46.4	4837	40.5	5780	42.8
24 to 28	10664	41.9	6000	43.3	2911	40.8	1740	39.4	5352	44.8	5312	39.4
≥28	4171	16.4	2467	17.8	1067	15.0	630	14.3	1765	14.8	2406	17.8
Number of Antihyperglycemic Agents												
None	1870	7.3	1036	7.5	491	6.9	339	7.7	932	7.8	938	6.9
Monotherapy	11776	46.3	6102	44.0	3418	47.9	2235	50.6	5547	46.4	6229	46.1
Dual therapy	8970	35.2	4977	35.9	2496	35.0	1479	33.5	4133	34.6	4837	35.8
Triple therapy or more	2838	11.1	1740	12.6	728	10.2	368	8.3	1343	11.2	1495	11.1
Smoking												
No	21283	83.6	10730	77.4	6404	89.8	4109	92.9	8067	67.5	13216	97.9
Yes	4171	16.4	3125	22.6	729	10.2	312	7.1	3888	32.5	283	2.1
Drinking												
No	23568	92.6	12407	89.5	6829	95.7	4287	97.0	10131	84.7	13437	99.5
Yes	1886	7.4	1448	10.5	304	4.3	134	3.0	1824	15.3	62	0.5
Physical activities												
Frequent/PRN	16216	63.7	9323	67.3	4797	67.3	2085	47.2	7600	63.6	8616	63.8
No exercise	9238	36.3	4532	32.7	2336	32.7	2336	52.8	4355	36.4	4883	36.2
Number of Antihyperglycemic Agents												
None	1870	7.3	1036	7.5	491	6.9	339	7.7	932	7.8	938	6.9
Monotherapy	11776	46.3	6102	44.0	3418	47.9	2235	50.6	5547	46.4	6229	46.1

T2D, type 2 diabetes mellitus; HbA1c, glycated hemoglobin; SBP, systolic blood pressure; DBP, diastolic blood pressure; LDL-c, low-density lipoprotein cholesterol; HDL-c, high-density lipoprotein cholesterol; BMI, body mass index; eGFR, estimated glomerular filtration rate.

The overall median HbA1c value was 7.10%. The percentage of patients with HbA1c values of <7%, 7% to <8%, 8% to <9%, and ≥9% was 44.8%, 21.5%, 12.5%, and 21.0%, respectively. The overall median SBP and DBP values were 130 mmHg and 80 mmHg, respectively. SBP and DBP values were similar across all age and gender groups.

The overall median total cholesterol, LDL-c, HDL-c values, and triglycerides were 4.89, 2.76, 1.23, and 1.56 mmol/L, respectively. Total cholesterol, LDL-c, and triglyceride values decreased with increasing age, and males had lower total cholesterol, LDL-c, HDL-c, and triglyceride values compared to females.

The overall median eGFR value was 88.97 ml/min/1.73m², which decreased with increasing age and was higher in males than in females. The percentage of patients with eGFR values ≥90, 60 to <90, 45 to <60, 30 to <45, 15 to <30, and <15 ml/min/1.73m² was 43.3%, 29.2%, 9.0%, 3.8%, 1.7%, and 1.8%, respectively.

A total of 10,617 (41.7%) had a BMI <24 kg/m², 10,664 (41.9%) had a BMI of 24-<28 kg/m², and 4,171 (16.4%) had a BMI ≥28 kg/m². More than 50% of patients were overweight or obese across all age and gender groups.

Regarding therapy information, 1,870 (7.3%) did not receive any antihyperglycemic therapy, 11,776 (46.3%) received monotherapy, 8,970 (35.2%) received dual therapy, and 2,838 (11.1%) received triple therapy or more. These patterns were similar across all age and gender groups.

Among all included patients, 4,171 (16.4%) were smokers, and 1,886 (7.4%) were drinkers. The proportion of smoking and drinking patients decreased with increasing age and was higher in male patients compared to females. A total of 16,216 (63.7%) patients engaged in frequent physical activity (3 times per week), while 9,238 (36.3%) patients did not. The proportion of patients with frequent physical activity significantly decreased in patients >75 years old.

### Prevalence of comorbidities with age and gender differences

A total of 20,309 (79.8%) patients in the study exhibited at least one comorbid condition. Among them, 24.6% had two comorbidities, 15.6% had three, and 11.6% had four or more comorbidities. Females with T2DM had a higher overall percentage of comorbidities compared to males (42.7% vs. 37.1%). The longer duration of T2DM, the more proportion of patients had comorbidities. For patients with a T2DM duration of less than 1 year, between 1 and 5 years, more than 5 to 10 years, and over 10 years, the percentage of patients with any comorbidity was 6.3%, 24.4%, 19.1% and 29.9%, respectively (**Table 2**).

**Table 2:** Summary of patients with different number of comorbid conditions, overall, and by duration of diabetes.

	With comorbidities		With 1 comorbidity		With 2 comorbidities		With 3 comorbidities		With ≥4 comorbidities	
	N	% (95% CI)	N	% (95% CI)	N	% (95% CI)	N	% (95% CI)	N	% (95% CI)
**Overall**	20309	79.8 (79.3–80.3)	7121	28.0 (27.4–28.5)	6262	24.6 (24.1–25.1)	3964	15.6 (15.1–16.0)	2962	11.6 (11.2–12.0)
Gender										
Male	9438	37.1 (36.5–37.7)	3370	13.2 (12.8–13.7)	2922	11.5 (11.1–11.9)	1856	7.3 (7.0–7.6)	1290	5.1 (4.8–5.3)
Female	10871	42.7 (42.1–43.3)	3751	14.7 (14.3–15.2)	3340	13.1 (12.7–13.5)	2108	8.3 (7.9–8.6)	1672	6.6 (6.3–6.9)
Duration of T2DM										
<1 year	1614	6.3 (6.0–6.6)	733	2.9 (2.7–3.1)	541	2.1 (2.0–2.3)	254	1.0 (0.9–1.1)	86	0.3 (0.3–0.4)
1–5 years	6219	24.4 (23.9–25.0)	2634	10.3 (10.0–10.7)	1979	7.8 (7.5–8.1)	1066	4.2 (3.9–4.4)	540	2.1 (2.0–2.3)
>5 to 10 years	4864	19.1 (18.6–19.6)	1733	6.8 (6.5–7.1)	1538	6.0 (5.8–6.3)	939	3.7 (3.5–3.9)	654	2.6 (2.4–2.8)
>10 years	7612	29.9 (29.3–30.5)	2021	7.9 (7.6–8.3)	2204	8.7 (8.3–9.0)	1705	6.7 (6.4–7.0)	1682	6.6 (6.3–6.9)

Among patients with T2DM, the most prevalent comorbid conditions in T2DM patients were HTN in 59.9%, overweight/obesity in 58.3%, hyperlipidemia in 42.0%, retinopathy in 16.5%, neuropathy in 15.2%, CVD in 14.9%, and renal disease in 14.4% (**Table 3**).

**Table 3:** Summary of comorbidities of interest in patients with T2DM, overall, by age and by gender.

Comorbidities	Overall		<65 years		65–74 years		≥75 years		Chi-square	P value	Male		Female		Chi-square	P value
	N	% (95% CI)	N	% (95% CI)	N	% (95% CI)	N	% (95% CI)			N	% (95% CI)	N	% (95% CI)		
**CVD**	3788	14.9 (14.4–15.3)	1351	9.8 (9.3–10.3)	1326	18.6 (17.7–19.5)	1103	24.9 (23.7–26.2)	719.29	**<.0001**	1719	14.4 (13.8–15.0)	2069	15.3 (14.7–15.9)	4.50	**0.03**
**CBD**	2574	10.1 (9.7–10.5)	833	6.0 (5.6–6.4)	901	12.6 (11.9–13.4)	828	18.7 (17.6–19.9	668.83	**<.0001**	1284	10.7 (10.2–11.3)	1290	9.6 (9.1–10.1)	9.78	**0.00**
**PVD**	391	1.5 (1.4–1.7)	165	1.2 (1.0–1.4)	101	1.4 (1.2–1.7)	121	2.7 (2.3–3.3)	54.17	**<.0001**	230	1.9 (1.7–2.2)	161	1.2 (1.0–1.4)	22.41	**<.0001**
**Renal disease**	3673	14.4 (14.0–14.9)	1778	12.8 (12.3–13.4)	1100	15.4 (14.6–16.3)	782	17.7 (16.6–18.8)	72.40	**<.0001**	1911	16.0 (15.3–16.7)	1762	13.1 (12.5–13.6)	44.14	**<.0001**
**Retinopathy**	4196	16.5 (16.0–16.9)	1240	8.9 (8.5–9.4)	1686	23.6 (22.7–24.6)	1259	28.5 (27.2–29.8)	1299.15	**<.0001**	1608	13.5 (12.9–14.1)	2588	19.2 (18.5–19.8)	150.75	**<.0001**
**Neuropathy**	3861	15.2 (14.7–15.6)	2093	15.1 (14.5–15.7)	1076	15.1 (14.3–15.9)	683	15.4 (14.4–16.5)	0.35	0.84	1762	14.7 (14.1–15.4)	2099	15.5 (14.9–16.2)	3.24	0.07
**CHF**	333	1.3 (1.2–1.5)	89	0.6 (0.5–0.8)	120	1.7 (1.4–2.0)	119	2.7 (2.3–3.2)	122.39	**<.0001**	143	1.2 (1.0–1.4)	190	1.4 (1.2–1.6)	2.19	0.14
**Overweight/Obesity**	14835	58.3 (57.7–58.9)	8467	61.1 (60.3–61.9)	3978	55.8 (54.6–56.9)	2370	53.6 (52.1–55.1)	103.89	**<.0001**	7117	59.5 (58.6–60.4)	7718	57.2 (56.3–58.0)	14.49	**0.00**
**Overweight**	10664	41.9 (41.3–42.5)	6000	43.3 (42.5–44.1)	2911	40.8 (39.7–42.0)	1740	39.4 (37.9–40.8)	26.46	**<.0001**	5352	44.8 (43.9–45.7)	5312	39.4 (38.5–40.2)	76.42	**<.0001**
**Obesity**	4171	16.4 (15.9–16.8)	2467	17.8 (17.2–18.5)	1067	15.0 (14.1–15.8)	630	14.3 (13.3–15.3)	45.71	**<.0001**	1765	14.8 (14.1–15.4)	2406	17.8 (17.2–18.5)	43.32	**<.0001**
**HTN**	15237	59.9 (59.3–60.5)	6875	49.6 (48.8–50.5)	4948	69.4 (68.3–70.4)	3380	76.5 (75.2–77.7)	1379.16	**<.0001**	6779	56.7 (55.8–57.6)	8458	62.7 (61.8–63.5)	93.48	**<.0001**
**Hyperlipidemia**	10688	42.0 (41.4–42.6)	5988	43.2 (42.4–44.0)	2937	41.2 (40.0–42.3)	1753	39.7 (38.2–41.1)	20.45	**<.0001**	5097	42.6 (41.8–43.5)	5591	41.4 (40.6–42.3)	3.86	0.05

CVD, cardiovascular disease; CBD, cerebrovascular disease; PVD, peripheral vascular disease; eGFR, estimated glomerular filtration rate; CHF, congestive heart failure; HTN, hypertension. Bold values denote statistical significance at the P < 0.05 level.

These common comorbid conditions were consistently observed across all age subgroups. The prevalence of comorbidities such as CVD, CBD, PVD, renal disease, retinopathy, CHF, and HTN increased uniformly with advancing age. Conversely, the prevalence of overweight/obesity, and hyperlipidemia was highest among younger patients. In patients under the age of 65 years, being overweight/obese was the most frequently observed condition, surpassing HTN. The prevalence of neuropathy seemed to remain relatively consistent across all age groups (**Table 3**).

Moreover, there were notable differences in the prevalence of certain comorbid conditions based on gender. In particular, CBD, PVD, renal disease, and overweight were more prevalent in men compared to women. On the other hand, CVD, retinopathy, obesity, and HTN were less frequent in male patients compared to their female counterparts (**Table 3**).

### Co-prevalence of comorbidities

Among the comorbid conditions studied, the highest co-prevalence was found between overweight/obesity and HTN, with a rate of 37.6%. The second highest co-prevalence was observed between HTN and hyperlipidemia, with a rate of 29.8%. Following closely, the co-prevalence of overweight/obesity and hyperlipidemia was 27.3%. Additionally, the study found that HTN coexisted with cardiovascular disease (CVD) in 12.6% of cases, with retinopathy in 12.1% of cases, and with renal disease in 11.3% of cases (**Table 4**).

**Table 4:** Co-prevalence of comorbidities (and eGFR stage) in patients with T2DM.

Overall population N=25206	CVD	CBD	PVD	Renal disease	Retinopathy	Neuropathy	CHF	Overweight/Obesity	Overweight	Obesity	HTN	Hyperlipidemia	eGFR≥90	60≤eGFR<90	30≤eGFR<60	15≤eGFR<30	eGFR<15
**CVD**	3788 (14.9)	603 (2.4)	89 (0.3)	882 (3.5)	1074 (4.2)	811 (3.2)	218 (0.9)	2419 (9.5)	1686 (6.6)	733 (2.9)	3200 (12.6)	2416 (9.5)	1288 (5.1)	1212 (4.8)	620 (2.4)	112 (0.4)	100 (0.4)
**CBD**		2574 (10.1)	63 (0.2)	621 (2.4)	796 (3.1)	574 (2.3)	74 (0.3)	1507 (5.9)	1074 (4.2)	433 (1.7)	2155 (8.5)	1369 (5.4)	877 (3.4)	870 (3.4)	445 (1.7)	68 (0.3)	48 (0.2)
**PVD**			391 (1.5)	130 (0.5)	104 (0.4)	183 (0.7)	10 (<0.1)	212 (0.8)	134 (0.5)	78 (0.3)	285 (1.1)	196 (0.8)	155 (0.6)	120 (0.5)	66 (0.3)	15 (0.1)	17 (0.1)
**Renal disease**				3673 (14.4)	895 (3.5)	979 3.8)	125 (0.5)	2286 (9.0)	1562 (6.1)	724 (2.8)	2879 (11.3)	2047 (8.0)	1183 (4.6)	962 (3.8)	735 (2.9)	267 (1.0)	290 (1.1)
**Retinopathy**					4196 (16.5)	1051 (4.1)	116 (0.5)	2355 (9.3)	1667 (6.5)	688 (2.7)	3079 (12.1)	2070 (8.1)	1385 (5.4)	1455 (5.7)	724 (2.8)	108 (0.4)	115 (0.5)
**Neuropathy**						3861 (15.2)	83 (0.3)	2235 (8.8)	1560 (6.1)	675 (2.7)	2499 (9.8)	2000 (7.9)	1808 (7.1)	1164 (4.6)	504 (2.0)	98 (0.4)	76 (0.3)
**CHF**							333 (1.3)	209 (0.8)	141 (0.6)	68 (0.3)	299 (1.2)	188 (0.7)	76 (0.3)	81 (0.3)	97 (0.4)	21 (0.1)	28 (0.1)
**Overweight/Obesity**								14835 (58.3)	NA	NA	9573 (37.6)	6954 (27.3)	6511 (25.6)	4242 (16.7)	1827 (7.2)	262 (1.0)	227 (0.9)
**Overweight**									10664 (41.9)	NA	6606 (26.0)	4831 (19.0)	4614 (18.1)	3108 (12.2)	1292 (5.1)	194 (0.8)	165 (0.6)
**Obesity**										4171 (16.4)	2967 (11.7)	2123 (8.3)	1897 (7.5)	1134 (4.5)	535 (2.1)	68 (0.3)	62 (0.2)
**HTN**											15237 (59.9)	7573 (29.8)	5797 (22.8)	4620 (18.2)	2265 (8.9)	382 (1.5)	424 (1.7)
**Hyperlipidemia**												10688 (42.0)	4643 (18.2)	3217 (12.6)	1278 (5.0)	195 (0.8)	173 (0.7)
**eGFR≥90**													11025(43.3)	NA	NA	NA	NA
**60≤eGFR<90**														7429 (29.2)	NA	NA	NA
**30≤eGFR<60**															3236 (12.7)	NA	NA
**15≤eGFR<30**																439 (1.7)	NA
**eGFR<15**																	460 (1.8)

Data were expressed as No. (%) of patients.

CVD, cardiovascular disease; CBD, cerebrovascular disease; PVD, peripheral vascular disease; eGFR, estimated glomerular filtration rate; CHF, congestive heart failure; HTN, hypertension.

Renal disease was defined as prior medical history of renal disease including diabetic nephropathy and other chronic kidney diseases (CKD stage 3–5).

The eGFR stage information was derived from previous hospital outpatient examinations.

When examining different age groups, the prevalence of comorbidity combinations with a co-occurrence rate of ≥10% increased with age. However, the three most common comorbidity combinations with a prevalence of ≥20% remained consistent across all age groups, namely overweight/obesity and hypertension, hypertension and hyperlipidemia, and overweight/obesity and hyperlipidemia (**Table 5**).

**Table 5:** Co-prevalence (%) of comorbidities (and eGFR stage) in patients with T2DM, by age group and by sex group.

	CVD	CBD	PVD	Renal disease	Retinopathy	Neuropathy	CHF	Overweight/Obesity	Overweight	Obesity	HTN	Hyperlipidemia	eGFR≥90	60≤eGFR<90	30≤eGFR<60	15≤eGFR<30	eGFR<15
Age group <65 years (N=13855) N=13855																	
CVD	9.8	1.1	0.2	2.1	1.6	2.2	0.4	6.8	4.5	2.3	7.8	6.8	4.5	2.7	0.8	0.2	0.3
CBD		6.0	0.2	1.3	1.0	1.5	0.1	3.9	2.8	1.1	4.7	3.7	2.7	1.7	0.6	0.1	0.1
PVD			1.2	0.4	0.3	0.6	<0.1	0.7	0.4	0.3	0.9	0.7	0.5	0.3	0.2	0.1	0.1
Renal disease				12.8	2.0	3.1	0.3	8.3	5.4	2.9	9.3	7.7	5.6	3.2	1.6	0.7	1.0
Retinopathy					8.9	2.7	0.1	5.1	3.6	1.5	5.6	4.6	4.1	2.4	1.0	0.3	0.3
Neuropathy						15.1	0.1	9.0	6.1	2.9	8.2	8.0	8.9	3.8	1.1	0.3	0.3
CHF							0.6	0.4	0.3	0.1	0.6	0.5	0.2	0.1	0.1	0.1	0.1
Overweight/Obesity								61.1	NA	NA	33.8	29.9	33.7	14.9	3.9	0.7	0.8
Overweight									43.3	NA	22.5	20.4	23.5	10.8	2.7	0.5	0.6
Obesity										17.8	11.4	9.5	10.2	4.1	1.1	0.2	0.2
HTN											49.6	26.7	24.5	12.6	4.2	1.0	1.4
Hyperlipidemia												43.2	23.5	10.9	2.8	0.6	0.6
eGFR≥90													54.4	NA	NA	NA	NA
60≤eGFR<90														24.6	NA	NA	NA
30≤eGFR<60															6.8	NA	NA
15≤eGFR<30																1.2	NA
eGFR<15																	1.6
Age group 65–74 years (N=7133)																	
CVD	18.6	3.0	0.3	4.2	6.3	4.1	1.2	11.8	8.5	3.3	16.0	11.8	5.5	6.8	3.1	0.4	0.4
CBD		12.6	0.3	2.9	4.3	2.7	0.4	7.2	5.1	2.1	10.7	6.5	4.1	4.6	2.1	0.3	0.2
PVD			1.4	0.4	0.5	0.6	<0.1	0.8	0.5	0.2	1.0	0.6	0.5	0.5	0.2	<0.1	0.1
Renal disease				15.4	4.6	4.6	0.6	9.4	6.8	2.6	12.6	8.1	3.7	4.6	3.4	1.2	1.4
Retinopathy					23.6	5.7	0.7	13.4	9.5	4.0	17.6	11.5	7.3	8.7	3.8	0.5	0.6
Neuropathy						15.1	0.5	8.5	6.2	2.3	11.3	7.9	5.4	5.4	2.6	0.4	0.3
CHF							1.7	1.2	0.7	0.5	1.5	1.0	0.4	0.5	0.5	0.1	0.1
Overweight/Obesity								55.8	NA	NA	41.5	25.4	17.9	19.1	9.4	1.3	1.0
Overweight									40.8	NA	29.5	18.1	13.2	14.3	6.6	0.9	0.7
Obesity										15.0	12.0	7.3	4.7	4.8	2.8	0.4	0.3
HTN											69.4	33.0	22.0	23.8	11.6	1.7	1.9
Hyperlipidemia												41.2	13.4	14.9	6.5	0.9	0.7
eGFR≥90													33.4	NA	NA	NA	NA
60≤eGFR<90														34.5	NA	NA	NA
30≤eGFR<60															16.4	NA	NA
15≤eGFR<30																2.0	NA
eGFR<15																	2.0
Age group ≥75 years (N=4421)																	
CVD	24.9	5.4	0.9	6.3	9.2	4.7	1.7	14.2	10.4	3.8	21.9	14.2	6.1	8.1	6.6	1.2	0.7
CBD		18.7	0.5	5.1	7.6	3.9	0.7	10.1	7.2	2.9	16.4	8.8	4.8	6.8	4.8	0.9	0.3
PVD			2.7	0.9	0.6	1.2	0.1	1.3	0.9	0.4	2.1	1.3	0.9	0.9	0.6	0.1	0.1
Renal disease				17.7	6.4	5.0	0.7	10.3	7.3	3.0	15.5	8.9	3.3	4.4	5.9	1.9	1.2
Retinopathy					28.5	6.3	1.1	15.4	11.0	4.4	23.4	13.6	6.6	11.2	7.2	0.7	0.6
Neuropathy						15.4	0.7	8.6	6.1	2.5	12.4	7.6	4.3	5.6	3.9	0.6	0.4
CHF							2.7	1.6	1.2	0.4	2.4	1.1	0.5	0.8	1.0	0.1	0.1
Overweight/Obesity								53.6	NA	NA	43.2	22.6	12.8	18.5	14.1	1.8	1.0
Overweight									39.4	NA	31.2	16.3	9.5	13.5	10.1	1.4	0.7
Obesity										14.3	12.0	6.3	3.3	5.0	4.0	0.3	0.2
HTN											76.5	34.1	18.7	26.6	19.3	2.7	2.1
Hyperlipidemia												39.7	9.8	14.5	9.6	1.2	0.9
eGFR≥90													25.0	NA	NA	NA	NA
60≤eGFR<90														35.4	NA	NA	NA
30≤eGFR<60															25.3	NA	NA
15≤eGFR<30																3.1	NA
eGFR<15																	2.2
Male (N=11955)																	
CVD	14.4	2.5	0.4	3.5	3.3	2.6	0.8	9.3	7.0	2.3	12.0	9.6	5.5	4.5	1.9	0.4	0.5
CBD		10.7	0.3	2.8	2.9	2.3	0.3	6.0	4.7	1.3	8.9	5.7	3.6	3.8	1.8	0.3	0.2
PVD			1.9	0.7	0.5	0.9	0.1	1.0	0.7	0.3	1.3	1.0	0.8	0.6	0.3	0.1	0.1
Renal disease				16.0	3.3	4.0	0.5	9.9	7.2	2.7	12.1	8.9	5.6	4.3	2.9	1.1	1.2
Retinopathy					13.5	3.3	0.4	7.4	5.6	1.7	9.6	6.1	4.8	4.8	1.8	0.4	0.5
Neuropathy						14.7	0.3	8.6	6.5	2.1	8.8	7.3	7.5	4.4	1.6	0.4	0.3
CHF							1.2	0.7	0.6	0.1	1.0	0.7	0.3	0.3	0.3	0.1	0.1
Overweight/Obesity								59.5	NA	NA	36.1	28.9	28.9	16.9	5.3	1.0	1.0
Overweight									44.8	NA	26.4	20.6	21.1	13.0	4.1	0.8	0.8
Obesity										14.8	9.8	8.3	7.8	3.9	1.2	0.2	0.3
HTN											56.7	28.7	23.3	17.5	6.8	1.4	1.8
Hyperlipidemia												42.6	20.5	12.8	4.0	0.6	0.6
eGFR≥90													47.8	NA	NA	NA	NA
60≤eGFR<90														29.1	NA	NA	NA
30≤eGFR<60															9.4	NA	NA
15≤eGFR<30																1.6	NA
eGFR<15																	1.9
Female (N=13499)																	
CVD	15.3	2.3	0.3	3.4	5.1	3.7	0.9	9.6	6.3	3.4	13.1	9.4	4.6	5.0	2.9	0.5	0.3
CBD		9.6	0.2	2.1	3.3	2.2	0.3	5.8	3.8	2.0	8.1	5.1	3.3	3.1	1.7	0.3	0.2
PVD			1.2	0.4	0.4	0.6	<0.1	0.7	0.4	0.3	0.9	0.6	0.4	0.4	0.2	<0.1	0.1
Renal disease				13.1	3.7	3.7	0.5	8.2	5.2	3.0	10.6	7.3	3.8	3.3	2.9	1.0	1.1
Retinopathy					19.2	4.9	0.5	10.9	7.4	3.5	14.3	9.9	6.0	6.6	3.8	0.4	0.4
Neuropathy						15.5	0.4	8.9	5.8	3.1	10.7	8.4	6.8	4.8	2.3	0.4	0.3
CHF							1.4	0.9	0.6	0.4	1.3	0.8	0.3	0.3	0.5	0.1	0.1
Overweight/Obesity								57.2	NA	NA	38.9	25.9	22.6	16.4	8.9	1.1	0.8
Overweight									39.4	NA	25.6	17.5	15.5	11.5	6.0	0.8	0.6
Obesity										17.8	13.3	8.4	7.1	5.0	2.9	0.3	0.2
HTN											62.7	30.7	22.3	18.7	10.7	1.6	1.6
Hyperlipidemia												41.4	16.2	12.5	5.9	0.9	0.7
eGFR≥90													39.3	NA	NA	NA	NA
60≤eGFR<90														29.2	NA	NA	NA
30≤eGFR<60															15.6	NA	NA
15≤eGFR<30																1.8	NA
eGFR<15																	1.7

Data were expressed as percentage (%) of patients.

CVD, cardiovascular disease; CBD, cerebrovascular disease; PVD, peripheral vascular disease; eGFR, estimated glomerular filtration rate; CHF, congestive heart failure; HTN, hypertension.

Renal disease was defined as prior medical history of renal disease including diabetic nephropathy and other chronic kidney diseases (CKD stage 3–5).

The eGFR stage information was derived from previous hospital outpatient examinations.

Similarly, when assessing gender differences among all patients, the top three comorbidity combinations with co-prevalence of ≥ 20% remained unchanged: overweight/obesity and HTN, HTN and hyperlipidemia, and overweight/obesity and hyperlipidemia (**Table 5**).

## Discussion

This study comprehensively evaluated the prevalence and co-prevalence of comorbidities among Chinese adult patients with T2DM using data from the 3B Study database, which was one of the largest-scale retrospective analyses to date within the Chinese population on this subject This study revealed that approximately 80% of Chinese adult T2DM patients had at least one comorbidity. The most prevalent comorbid conditions were HTN, overweight/obesity, and hyperlipidemia, affecting 59.9%, 58.3%, and 42.0% of the study population, respectively. Furthermore, variations in the prevalence of comorbidities were observed based on gender and age, with the most prevalent co-prevalence being between overweight/obesity and HTN, followed by HTN and hyperlipidemia, as well as overweight/obesity and hyperlipidemia.

The findings in this secondary analysis align with previous global research, indicating a substantial burden of comorbidities among T2DM patients (7–10). While proportions of T2DM-related comorbidities may vary across regions, we found that 79.8% of the overall population had at least one comorbidity. This prevalence is similar to the 73.1% reported in a previous study in 2016 (27), higher than the 65.2% observed in 2013 (28), and lower than the 93.7% reported in 2019 (15) in other studies in China. Notably, a previous study in the USA even recorded a prevalence of 97.5% (8).

Studies conducted in the USA have shown that more than 90% of diabetic patients exhibit two or more chronic conditions (8, 29, 30), and this figure reaches 75% in the UK (31). In contrast, previous domestic data for Chinese T2DM patients have been limited. A study conducted in a tertiary hospital in an eastern Chinese province found that up to 77.5% of T2DM patients had two or more comorbidities, but this study reports a slightly lower figure of 51.8% for patients with two or more comorbidities (8). It’s important to consider that different healthcare systems and patient demographics, such as age, sex, education, occupation, marital status, and residence, can influence the proportion of comorbid conditions in various populations (15). The 3B study’s large sample size and nationwide representation likely provide a more accurate reflection of the situation in China. However, it’s noteworthy that the real situation may be underestimated, as 89.5% of our patients reside in urban areas with better access to healthcare systems. In addition, the 3B study exclusively enrolled outpatients, potentially excluding patients with more severe medical conditions who may have required hospitalization.

Age and duration of T2DM are consistent determinants of comorbidities in T2DM patients (8, 32). It’s unsurprising that comorbid conditions increased from 6.3% in patients with less than 1 year of T2DM duration to 29.9% in those with more than 10 years of T2DM duration in this study. Most comorbidities were positively associated with age, consistent with previous reports that the burden of comorbidity tends to increase in older age groups (8, 32). Notably, we found that overweight, obesity, and hyperlipidemia were more commonly observed in younger diabetic Chinese patients, consistent with previous findings suggesting that the odds of obesity decrease with age in diabetic patients, possibly due to unintentional weight loss resulting from illness or the aging process itself (33, 34). Evidence also suggests that obesity in the elderly might not carry the same risks as in younger individuals and could, in certain aspects, even be protective (34). In terms of gender differences, the present study revealed that CVD, HTN and retinopathy were more common in females. However, it’s essential to consider that female diabetic patients in this study had a longer duration of DM, which might overestimate the prevalence of these comorbidities. Furthermore, contrary to some international studies reporting a higher prevalence of CVD in males, our results align with the concept that the risk of cardiovascular events in females with diabetes is 25–50% higher than in males (32, 35, 36). The pathogenesis appears multifactorial, involving sex-based genetic and biological factors, gender-related cultural and environmental disparities, as well as documented variations in the diagnosis, management, and treatment of T2DM and CVD between women and men (37, 38). Diabetic retinopathy is one of the most frequent and serious microvascular complications in T2DM and it remains ambiguous which sex is more susceptible to it. This study confirmed a large-scale twelve-province cross-sectional study in China, which demonstrated that females had a higher prevalence of diabetic retinopathy than males in T2DM patients (39). Additionally, previous research suggested that only females over the age of 60 years had a higher prevalence of diabetic retinopathy than males, indicating a potential protective role of estrogen in the occurrence and development of DR (39–41).

HTN, overweight/obesity and hyperlipidemia, individually or in combination, consistently rank as the three most common comorbidities in T2DM patients, sharing overlapping risk factors that lead to common pathways of complications (42). An international comparative review exploring hypertension and obesity in T2DM demonstrated that HTN rates were typically high in all regions, with most studies reporting HTN rates above 50% and obesity rates exceeding 30%. This is in line with findings observed in this study, where HTN prevalence reached 59.9% and overweight/obesity reached 58.3% (43). Current clinical practice guidelines from organizations like the ADA and CDS recommend assessing comorbidities such as CVD, renal disease, retinopathy, and neuropathy to help individualize targets for glycemia, blood pressure, and lipids, as well as select specific glucose-lowering medications, antihypertensive medications, and statin treatment intensity (11, 12).

The coexistence of multiple comorbidities in T2DM patients is associated with a higher risk of mortality (44). DM is the primary contributor of CKD as well as numerous macrovascular and microvascular complications (45). CKDs and CVDs represent two major causes of morbidity, impacting 20–40% of diabetic patients (46–48). While integrated diabetes management has reduced T2DM comorbidities in recent years, this trend differs in China. Studies in the USA, Hong Kong, and Sweden show declining T2DM comorbidity rates, but China saw an increase in microvascular and macrovascular comorbidities among T2DM inpatients from 2013 to 2017, likely due to inadequate integrated comorbidity management and suboptimal patient compliance (49). Additionally, multiple comorbidities place an added financial burden on T2DM patients, and challenge physicians in overall disease management (50). Prescribing treatment becomes intricate, considering the risk-benefit balance of polypharmacy (51). Regularly assessing comorbidities is crucial when selecting appropriate diabetes treatment regimens. Enhanced comprehension of the comorbidity landscape within the T2DM population enables the identification of more defined study cohorts for novel therapies, such as GLP-1 receptor agonists (GLP-1 RAs) and sodium-glucose co-transporter-2 (SGLT2) inhibitors. These treatments not only improve glycemic control, but also confer benefits in terms of blood lipid management, weight reduction, renal protection and cardiovascular risk reduction (52).

While this study contributes valuable insights into the prevalence and co-prevalence of comorbidities among Chinese T2DM patients, it is important to acknowledge its limitations. First, this was a retrospective, observational, and cross-sectional study without follow-up. Potential selection bias might exist due to the inclusion of patients, and it’s possible that some T2DM patients developed comorbidities at a later date. In addition, this study only included participants with DM duration exceeding six months, comprising predominantly elderly individuals with a median age of 63. The selection bias may compromise the validity of this study. Second, the study did not include some potential confounding variables, such as socioeconomic status, or access to healthcare, which may lead to biased estimates of the prevalence and co-prevalence of comorbidities in T2DM patients. Third, without exploring the temporal links between T2DM diagnosis and the development of comorbidities, the study may not provide a comprehensive understanding of how these conditions evolve over time. Fourth, the generalizability of the findings is limited because this study relied on secondary analysis of data from the existing 3B study, which was conducted between August 2010 and March 2011. Therefore, the study may not fully represent the current situation of Chinese T2DM population. Despite these limitations, the study’s considerable sample size and diverse geographical representation enhance its external validity.

## Conclusions

In conclusion, this study provides robust evidence that the majority of Chinese adult T2DM patients experience multiple comorbidities. These findings highlight the necessity for comprehensive patient management strategies that integrates not only glycemic control but also the management of associated comorbidities. Further research, including prospective studies and interventional trials, will be essential to elucidate the optimal treatment strategies for this complex patient population.

## Data availability statement

The raw data supporting the conclusions of this article will be made available by the authors, without undue reservation.

## Ethics statement

The studies involving humans were approved by ethics committee of Peking University People’s Hospital. The studies were conducted in accordance with the local legislation and institutional requirements. The ethics committee/institutional review board waived the requirement of written informed consent for participation from the participants or the participants’ legal guardians/next of kin because the requirement for informed consent was waived due to the retrospective study design.

## Author contributions

QJ: Conceptualization, Data curation, Formal analysis, Investigation, Methodology, Project administration, Software, Supervision, Writing – review & editing. SC: Conceptualization, Data curation, Formal analysis, Investigation, Methodology, Software, Writing – original draft, Writing – review & editing. RZ: Conceptualization, Formal analysis, Methodology, Software, Writing – review & editing. JL: Data curation, Writing – review & editing. YZ: Funding acquisition, Investigation, Writing – review & editing. SR: Investigation, Writing – review & editing.
